# Clinical significance of structural remodeling concerning substrate characteristics and outcomes in arrhythmogenic right ventricular cardiomyopathy

**DOI:** 10.1016/j.hroo.2022.04.007

**Published:** 2022-05-05

**Authors:** Chin-Yu Lin, Fa-Po Chung, Yenn-Jiang Lin, Shih-Lin Chang, Li-Wei Lo, Yu-Feng Hu, Ta-Chuan Tuan, Tze-Fan Chao, Jo-Nan Liao, Ting-Yung Chang, Ling Kuo, Cheng-I Wu, Chih-Min Liu, Shin-Huei Liu, Jin-Long Huang, Yu-Cheng Hsieh, Shih-Ann Chen

**Affiliations:** ∗ Heart Rhythm Center, Division of Cardiology, Department of Medicine, Taipei Veterans General Hospital, Taipei, Taiwan; † Department of Medicine, National Yang Ming Chiao Tung University, School of Medicine, Taipei, Taiwan; ‡ Cardiovascular Center, Taichung Veterans General Hospital, Taichung, Taiwan

**Keywords:** Arrhythmogenic right ventricular cardiomyopathy, Scar, Right ventricular dysfunction, Ventricular arrhythmia, Ablation

## Abstract

**Background:**

The substrate and ablation outcome in arrhythmogenic right ventricular cardiomyopathy (ARVC) with or without right ventricular (RV) dysfunction is unclear.

**Objective:**

We aimed to investigate ablation outcome and substrate in ARVC patients with or without RV dysfunction.

**Methods:**

We retrospectively studied ARVC patients with (group 1) or without RV dysfunction (group 2) undergoing substrate mapping/ablation. Baseline characteristics and electrophysiological features were compared. The RV was divided into 7 prespecified segments. The scarred segment was defined as more than 50% of the area with bipolar scar. A multivariate regression analysis was performed to predict the risk of ventricular tachycardia (VT) recurrence.

**Results:**

A total of 106 patients were enrolled (57 in group 1 and 49 in group 2). There were more men (73.7% vs 32.7%, *P* < .05) in group 1 than group 2. Group 1 patients demonstrated larger abnormal substrate in both the endocardium (13.4 ± 14.7 cm^2^ vs 7.8 ± 5.4 cm^2^, *P* = .014) and in the epicardium (40.3 ± 27.7 cm^2^ vs 14.2 ± 12.6 cm^2^, *P* = .002) and had more scar in the inferior portion/tricuspid valve (TV) than group 2 patients. Twenty-five patients had recurrences of VT/ventricular fibrillation. After multivariate analysis, the presence of a superior TV scar in the endocardium predicted the recurrence in patients with sustained VT.

**Conclusion:**

The presence of RV dysfunction was associated with a larger abnormal substrate in the endocardium and epicardium of the RV. A scar involving the inferior portion and TV is associated with RV dysfunction. Scarring in the superior TV of the endocardium can predict recurrence despite catheter ablation.

**Key Findings**

▪ The presence of right ventricle (RV) dysfunction is associated with a larger abnormal substrate in the endocardium and epicardium of the RV.
▪ A scar involving the inferior portion and tricuspid valve is associated with RV dysfunction.
▪ Scarring in the superior tricuspid valve can predict recurrence despite catheter ablation.

## Introduction

Arrhythmogenic right ventricular cardiomyopathy (ARVC) is a type of inherited cardiomyopathy caused by mutations in the desmosomal proteins, which lead to the dysfunction of cellular adhesion molecules.^1^ ARVC is characterized by progressive fibrofatty replacement of the right ventricular (RV) myocardium creating a substrate for reentrant ventricular arrhythmias (VA).^2^, ^3^, ^4^ Catheter ablation has been established as an effective therapy for patients with ARVC and sustained ventricular tachycardia (VT). Combined epicardial and endocardial ablation may be required in some patients.^5^^,^^6^

End-stage RV failure or biventricular pump failure may develop in patients with long-standing disease.^7^, ^8^, ^9^ The involvement of epicardial substrate is usually more extensive than the endocardium, with an epicardium-to-endocardium progression pattern.^7^^,^^10^ The recent study suggested that patients with more advanced stage of ARVC tend to have less arrhythmic substrate in the epicardium owing to the progressive fibrofatty replacement at this level.^10^

The overall objective of this study was to determine if RV dysfunction affected ablation outcome. Furthermore, we tried to study the substrate properties in ARVC patients with or without RV dysfunction to investigate the scar pattern and the predictors of recurrence.

## Methods

### Study population

We enrolled patients diagnosed with ARVC based on the 2010 Revised Task Force Criteria,^11^ who had undergone endocardial and/or epicardial substrate mapping and radiofrequency catheter ablation for drug-refractory VA between 2013 and 2021. The indications for catheter ablation included the following: (1) individuals with recurrent sustained monomorphic VT refractory to antiarrhythmic drugs, and (2) symptomatic individuals with a high burden of ventricular ectopy and documented nonsustained VT refractory to antiarrhythmic drugs. The epicardial approach was considered for patients with ARVC.^12^ Endocardial approach was attempted initially for all the patients. Epicardial approach was indicated for patients with (1) unmatched endocardial substrate and VT exit, (2) lack of abnormal substrate in the endocardium, (3) failed endocardial ablation, and (4) incomplete VT circuit with endocardial mapping during VT.

All patients underwent 12-lead electrocardiogram (ECG), 24-hour Holter monitoring, transthoracic echocardiography, coronary arteriography, RV angiography, and electrophysiological evaluation. Magnetic resonance imaging (MRI) was performed in patients without contraindication. Endomyocardial biopsy was considered for all patients and performed after getting informed consent from the patients.

The patients were categorized into 2 groups according to the RV function, based on the Revised Task Force Criteria.^11^ Patients with RVEF ≤40% on MRI were classified as group 1 (RV dysfunction). In patients without interpretable MRI, RV angiography was used to confirm regional RV akinesia or dyskinesia with a decreased RVEF ≤40%. Patients with RVEF >40% on MRI were classified as group 2. In patients without interpretable MRI, RV angiography was used to confirm no RV dysfunction.

Baseline characteristics, echocardiographic and electrophysiological parameters, and substrate characteristics were compared between patients with and without RV dysfunction. The major/minor criteria of fibrofatty replacement, depolarization abnormalities, repolarization abnormalities, VA, and family history were based on the revised Task Force Criteria.^13^

This retrospective study was approved by the Institutional Review Board. The research reported in this paper adhered to the Helsinki Declaration guidelines.

### Electrophysiological study

The details of the electrophysiological study, substrate mapping, and ablation strategies were described in our previous work.^2^ After obtaining informed consent, we performed a standardized electrophysiological study for all patients under fasting and sedated status. All antiarrhythmic drugs except amiodarone were discontinued for at least 5 half-lives prior to radiofrequency catheter ablation.^2^ Rapid ventricular pacing and/or programmed stimulation up to 3 extrastimuli were performed from the RV apex and/or RV outflow tract (RVOT) to induce VT/ventricular fibrillation (VF), with and without intravenous isoproterenol (1–5 μg/min). The QRS morphologies and cycle lengths (CL) of spontaneous and/or induced VTs were compared with those of clinically documented VTs.

### Three-dimensional electroanatomic mapping, and ablation

Bipolar scar/low-voltage zone (LVZ) were defined by<0.5 and <1.5 mV, respectively. The unipolar LVZ was considered once unipolar voltage was less than 5.5 mV.^14^ The average bipolar or unipolar median voltage was calculated. The area of the scar, LVZ, and area of abnormal substrate (defined as electrogram with late potential or an abnormal electrogram inscribed within the QRS, or continuous fragmented potentials)^15^ were measured using the standard surface area measurement tool on the navigation system. When multiple areas with confluent low voltages were present, the aggregate area from the individual regions of interest was calculated. Each value of percentage was calculated by dividing the total endocardial RV area or epicardial RV area. To achieve homogeneously detailed maps, the fill threshold was set to 10 mm in areas with normal voltages and to 5 mm in areas with low-voltage amplitude, as in our previous publication.^2^

Once the stable VT was induced, activation mapping and/or entrainment mapping of stable VT was performed to localize the VT isthmus. A substrate-based ablation strategy targeting the late and fractionated electrograms within or surrounding the scar/LVZ was performed in all patients.

Successful ablation was defined as the absence of any spontaneous or inducible VA using the same stimulation protocol at the end of the procedure, with and without isoproterenol.^2^ Partial success was defined as the presence of either spontaneous or inducible nonclinical VA after ablation, while failed ablation was considered for those with inducible clinical VAs.

### Scar distribution

Based on electroanatomic mapping, the epicardial and endocardial free wall of the RV was categorized into 7 distinct anatomical RV segments based on our previous publication.^16^ The right ventricle was also categorized into 7 distinct anatomical RV segments, including RVOT (from the pulmonic valve to the top of the tricuspid valve), superior tricuspid valve (TV; 2 cm anterior to the valve, superior portion), inferior TV (2 cm anterior to the valve, inferior portion), superior free wall (the other superior portion of the RV free wall), inferior free wall (the other inferior portion of the RV free wall), anterior wall, and apex. The segment was defined as a scarred segment if more than 50% of the area in the prespecified segments demonstrated a bipolar voltage of less than 0.5 mV.

### Follow-up and recurrences of VA

Patients underwent regular follow-up at 1, 3, and 6 months after ablation in the first year and every 3–6 months thereafter. Implantable cardioverter-defibrillator (ICD) interrogation, ECG, and Holter monitoring were performed every 3 or 6 months. The cause of mortality during follow-up was classified into cardiovascular-caused mortality or non–cardiovascular-caused mortality according to the death diagnosis. Recurrent VAs were defined as recurrent sustained VT/VF.^17^ The events of appropriate ICD therapy included antitachycardia pacing and defibrillation. In the patients without ICD, the events were defined as sustained VT/VF in the Holter monitoring, surface ECG, ECG strips, or automated external defibrillator recording. These events were reviewed by at least 2 electrophysiologists.

### Statistical analysis

Continuous variables are expressed as mean ± standard deviation, while categorical variables are expressed as percentages. Differences between continuous variables were assessed using the Student *t* test, whereas categorical variables were compared using the χ^2^ test with or without Yates correction or Fisher exact test, as indicated. Statistical significance was set at *P* < .05. The Cox hazard ratio (HR) regression model included all parameters with significant differences (*P* < .05) between group 1 and group 2 in the baseline characteristics and electrophysiological study. All statistical analyses were performed using the Statistical Package for the Social Sciences (version 22.0; IBM Corporation, Armonk, NY).

## Results

### Baseline characteristics of patients with ARVC

One hundred and six patients (58 [54.7%] men; mean age, 46.6 ± 13.5 years) with a diagnosis of definite ARVC based on the 2010 Revised Task Force Criteria received endocardial and/or epicardial mapping and ablation. Patients were classified into 2 groups. Group 1 consisted of 57 patients with RV dysfunction, and group 2 comprised 49 patients without RV dysfunction. A total of 49 (46.2%) patients underwent endocardial and epicardial mapping. Drug-refractory sustained VT was documented in 81 patients (76.4%). A high burden of ventricular ectopy or nonsustained VT was documented in 25 (23.6%) symptomatic individuals. Of the total 106 patients, 68 patients agreed to and received endomyocardial biopsy. There were 72 patients offered genetic testing. MRI was performed in 91 patients (85.8%). Patients with RV dysfunction were classified as group 1, and other patients were classified as group 2. More patients in group 1 were male (42 [73.7%] vs 16 [32.7%], *P* < .001) and had decreased left ventricular ejection fraction (52.0% ± 7.8% vs 59.7% ± 8.5%, *P* < .001). There were no significant differences in the other baseline parameters, repolarization abnormalities, depolarization abnormalities, family history, and histopathologic evidence of fibrofatty infiltration between the 2 groups (Table 1). The major/minor criteria of fibrofatty replacement, depolarization abnormalities, repolarization abnormalities, VA, and family history were based on the revised Task Force Criteria.^13^ Forty-two (73.7%) and 26 (53.1%) patients underwent genetic analysis in group 1 and group 2, respectively. Seventeen (39.5%) and 8 (32.0%) patients in group 1 and group 2, respectively, demonstrated a mutation in the genes that were associated with ARVC, according to the Task Force criteria (*P* = .608).

**Table 1:** Comparison of baseline characteristics between arrhythmogenic right ventricular cardiomyopathy patients with and without right ventricular dysfunction

	Group 1 RV dysfunction (N = 57)	Group 2 No RV dysfunction (N = 49)	*P* value
Baseline characteristics			
Age, y	48.0 ± 14.6	44.6 ± 12.6	.202
Sex (male)	42 (73.7%)	16 (32.7%)	<.001
Hypertension	20 (35.1%)	11 (22.4%)	.200
Diabetes mellitus	5 (8.8%)	2 (4.1%)	.447
Documented sustained VT	48 (84.2%)	36 (73.5%)	.168
LVEF	52.0% ± 7.8%	59.7% ± 8.5%	<.001
LVEF <50%	17 (30.4%)	4 (8.3%)	.005
Preprocedural AAD			
Beta blocker	38 (66.7%)	26 (53.1%)	.169
Class I AAD	8 (14.0%)	17 (34.7%)	.021
Class III AAD	32 (56.1%)	19 (38.8%)	.083
Postprocedural AAD			
Beta blocker	30 (52.6%)	20 (40.8%)	.247
Class I AAD	10 (17.5%)	4 (8.2%)	.249
Class III AAD	22 (38.6%)	14 (28.6%)	.309
Fibrofatty replacement†			
Major	11 (19.3%)	12 (24.5%)	.263
Minor	14 (24.6%)	6 (12.2%)	
Depolarization abnormalities†			
Major	12 (21.1%)	3 (6.1%)	.083
Minor	42 (73.7%)	42 (85.7%)	
Repolarization abnormalities†			
Major	13 (22.8%)	5 (10.2%)	.207
Minor	23 (40.4%)	21 (42.9%)	
Ventricular arrhythmias†			
Major	21 (36.8%)	17 (34.7%)	.842
Minor	36 (63.2%)	32 (65.3%)	
Family history†			
Major	17 (29.8%)	8 (16.7%)	.114
Minor	1 (1.8%)	0 (0.0%)	
Procedure time (minutes)	215.9 ± 45.1	191.2 ± 35.5	.054
Ablation time (minutes)	45.6 ± 37.1	23.5 ± 21.4	.001
Epicardial approach	35 (61.4%)	14 (28.6%)	.001

AAD = antiarrhythmic drug; LVEF, left ventricular ejection fraction; RV = right ventricular; VT = ventricular tachycardia.

Results are mean ± SD or n (%).

† According to the 2010 Revised Task Force Criteria.^12^

### Electrophysiological study

The mean number of clinical VT was 1.1 ± 0.3 in group 1 and 1.0 ± 0.1 in group 2 (*P* = .234). The mean number of inducible VT was 1.7 ± 1.0 in group 1 and 1.2 ± 0.5 in group 2 (*P* = .006). The CL of clinical VT (323.5 ± 68.6 vs 286.9 ± 55.8 ms, *P* = .016) and induced VT (360.0 ± 84.4 vs 302.8 ± 55.1 ms, *P* = .001) was longer in group 1 in comparison to group 2.

Acute procedural success with noninducible VT was achieved in 48 (84.2%) and 44 (89.8%) patients of group 1 and group 2, respectively. Partial success with inducible nonclinical VT was achieved in 9 (15.8%) and 3 (6.1%) patients of group 1 and group 2, respectively. Failed procedure was noted with inducible clinical VT in 2 (4.1%) patients of group 2. The distribution of acute procedure outcome (acute procedural success, partial success, and failed procedure) was not significantly different (*P* = .100, Pearson χ^2^ test).

### Endocardial substrate characteristics

Table 2 shows the comparison of substrate characteristics of RV endocardium between group 1 and group 2 patients. The mean number of mapping points was 593 ± 479 points. Group 1 patients demonstrated the larger bipolar LVZ (35.1 ± 26.7 cm^2^ vs 23.1 ± 10.9 cm^2^, *P* = .027), bipolar scar (17.5 ± 13.8 cm^2^ vs 11.6 ± 10.9 cm^2^, *P* = .017), unipolar LVZ (66.5 ± 39.6 vs 45.9 ± 21.6 cm^2^, *P* = .002), and longer total activation time (155.0 ± 34.5 vs 140.1 ± 29.2 ms, *P* = .020) in comparison to the group 2 patients.

**Table 2:** Comparison of endocardial electrophysiological parameter between arrhythmogenic right ventricular cardiomyopathy patients with or without right ventricular dysfunction

	RV dysfunction (group 1, N = 57)	No RV dysfunction (group 2, N=49)	*P* value
RV endocardium			
Averaged bipolar voltage†	2.0 ± 0.8	2.2 ± 0.9	.213
Averaged unipolar voltage†	5.0 ± 1.5	5.4 ± 1.3	.141
Total activation time (ms)	155.0 ± 34.5	140.1 ± 29.2	.020
Bipolar low-voltage zone (cm^2^)	35.1 ± 26.7	23.1 ± 10.9	.027
Bipolar low-voltage zone, %	15.7 ± 11.7	12.0 ± 6.0	.044
Bipolar scar (cm^2^)	17.5 ± 13.8	11.6 ± 10.9	.017
Bipolar scar, %	8.3 ± 6.1	5.5 ± 4.1	.008
Unipolar low-voltage zone (cm^2^)	66.5 ± 39.6	45.9 ± 21.6	.002
Unipolar low-voltage zone, %	27.3 ± 13.4	21.1 ± 8.5	.007
Area with abnormal substrate (cm^2^)	13.4 ± 14.7	7.8 ± 5.4	.014
Scar distribution			
RVOT	32 (56.1%)	30 (61.2%)	.693
Superior free wall	8 (14.0%)	11 (22.4%)	.314
Inferior free wall	11 (19.3%)	0 (0.0%)	.001
Superior TV	21 (36.8%)	7 (14.3%)	.014
Inferior TV	29 (50.9%)	13 (26.5%)	.016

RV = right ventricle; RVOT = right ventricular outflow tract; TV = tricuspid valve.

Results are mean ± SD or n (%).

† The average of bipolar or unipolar median voltage.

Group 1 patients had more scarred segments in the inferior free wall (19.3% vs 0.0%, *P* = .001), superior TV (36.8% vs 14.3%, *P* = .014), and inferior TV (50.9% vs 26.5%, *P* = .016) in comparison to the group 2 patients.

### Epicardial substrate characteristics

Table 3 shows the comparison of substrate characteristics of the RV epicardium (n = 49) between group 1 and group 2 patients. The mean number of mapping points was 1528 ± 971 points. There was a similar bipolar LVZ and scar area between the 2 groups. Group 1 patients demonstrated the larger abnormal substrate (40.3 ± 27.7 cm^2^ vs 14.2 ± 12.6 cm^2^, *P* = .002) in comparison to the group 2 patients.

**Table 3:** Comparison of right ventricular epicardial substrate between patients with arrhythmogenic right ventricular cardiomyopathy with or without right ventricular dysfunction

	RV dysfunction (group 1, N = 57)	No RV dysfunction (group 2, N=49)	*P* value
RV epicardium			
Averaged bipolar voltage (mV)†	1.1 ± 0.4	1.5 ± 0.8	.076
Total activation time (ms)	207.9 ± 18.4	200.8 ± 42.2	.413
Bipolar low-voltage zone (cm^2^)	110.1 ± 52.2	86.8 ± 60.8	.185
Bipolar low-voltage zone, %	38.6 ± 23.1	27.3 ± 12.8	.092
Bipolar scar (cm^2^)	55.5 ± 30.1	45.0 ± 38.5	.312
Bipolar scar, %	18.8 ± 12.2	13.7 ± 7.8	.152
Area with abnormal potentials (cm^2^)	40.3 ± 27.7	14.2 ± 12.6	.002
Scar distribution			
RVOT	18 (51.4%)	13 (92.9%)	.008
Superior free wall	5 (14.3%)	2 (14.3%)	.999
Inferior free wall	22 (62.9%)	3 (21.4%)	.012
Superior TV	14 (40.0%)	10 (71.4%)	.062
Inferior TV	29 (82.9%)	6 (42.9%)	.012
Anterior wall	3 (8.6%)	0 (0.0%)	.548
Apex	4 (11.4%)	0 (0.0%)	.312

RV = right ventricle; RVOT = right ventricular outflow tract; TV = tricuspid valve.

Results are mean ± SD or n (%).

† The average of bipolar or unipolar median voltage.

Group 1 patients had more scarred segments in the inferior free wall (62.9% vs 21.4%, *P* = .012) and inferior TV (82.9% vs 42.9%, *P* = .012) in comparison to the group 2 patients. Conversely, group 1 patients had fewer scarred segments in the RVOT area (51.4% vs 92.9%, *P =* .008) than group 2 patients.

**Figure 1:**
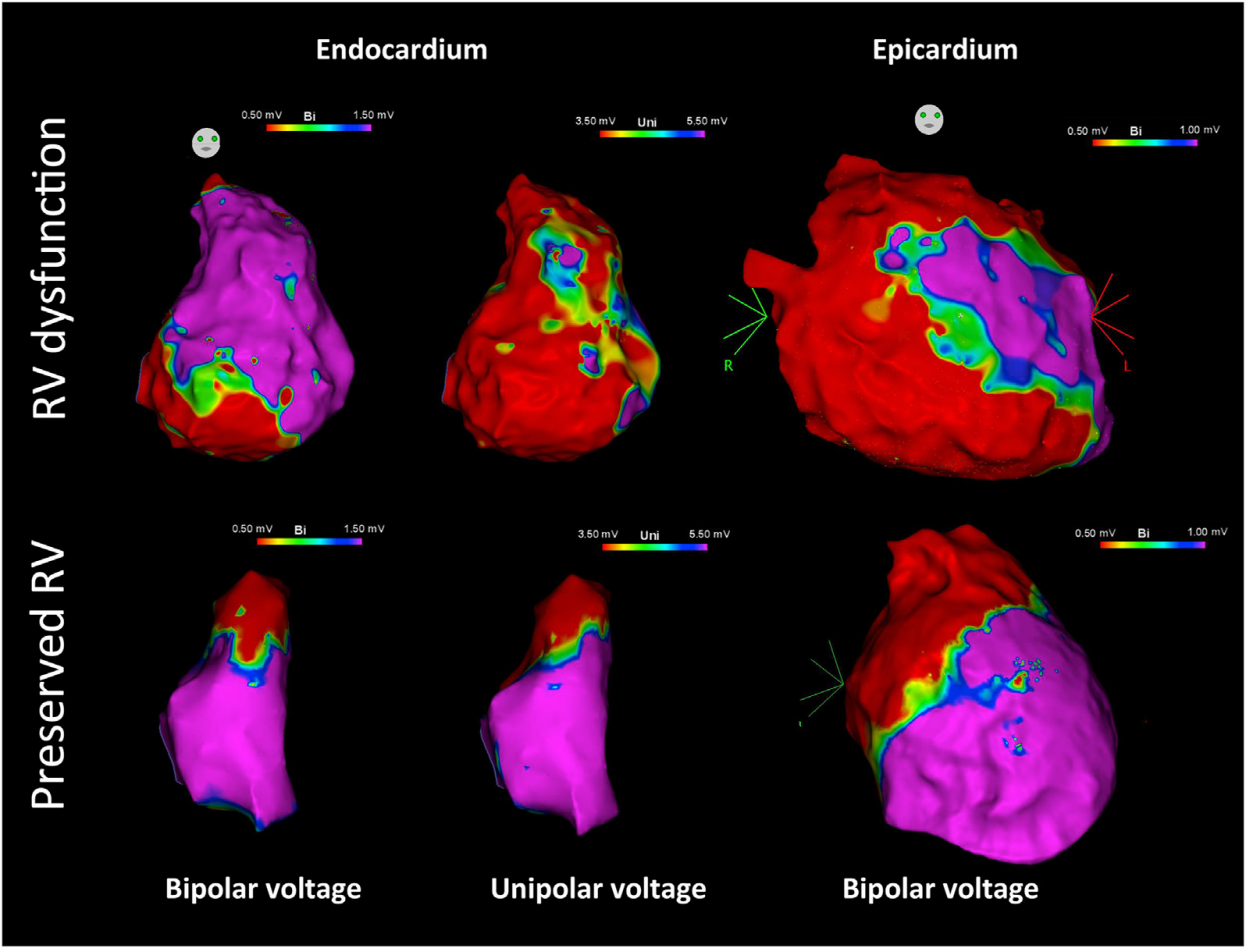
Arrhythmogenic right ventricular cardiomyopathy (ARVC) patients with and without right ventricle (RV) dysfunction. **Top:** An example of an ARVC patient with severe RV dysfunction. The endocardial bipolar voltage map (left image) shows a dense scar in the inferior tricuspid valve (TV) and inferior free wall. The endocardial unipolar voltage map (middle image) shows an extensive low-voltage zone in the TV and free wall. The epicardial bipolar voltage map shows extensive scarring in the right ventricular outflow tract (RVOT), entire TV, and inferior free wall area. **Bottom:** An example of an ARVC patient without RV dysfunction. The endocardial bipolar voltage map (left image) shows a dense scar in the RVOT area. The endocardial unipolar voltage map (middle image) shows a comparable low-voltage zone in the RVOT area. The epicardial bipolar voltage map shows extensive scarring in the RVOT and superior TV area.

**Figure 2:**
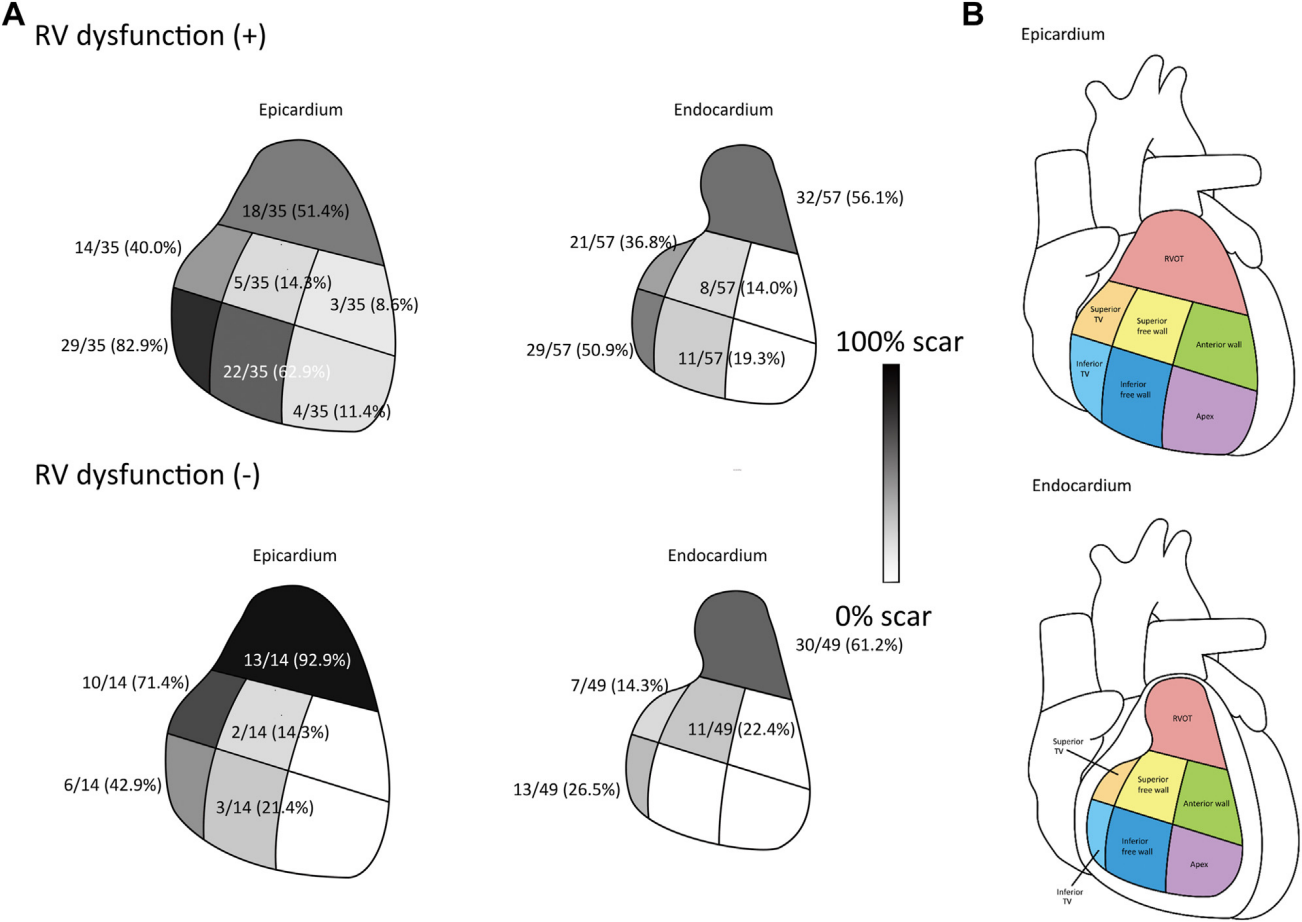
The diverse scar distribution pattern in arrhythmogenic right ventricular cardiomyopathy patients with and without right ventricle (RV) dysfunction. **A:** Upper row: In patients with RV dysfunction, the scar pattern is illustrated (left image: epicardium; right image: endocardium). Lower row: In patients without RV dysfunction, the scar pattern is illustrated (left image: epicardium; right image: endocardium). **B:** The segmentation of the epicardium (top image) and endocardium (bottom image) of right ventricle. The details and statistical results are summarized in Tables 2 and 3.

Figure 1 shows an example of epicardial/endocardial bipolar voltage mapping for groups 1 and 2, respectively. Figure 2 summarizes the distribution of the scarred segment in the RV epicardium and endocardium from groups 1 and 2.

### Follow-up

After a mean follow-up period of 45.3 ± 28.5 months, 3.5% (2/57) and 2.0% (1/49) of the patients died of noncardiovascular diseases in group 1 and group 2, respectively. A total of 35.1% (20/57) and 10.2% (5/49) of the patients had recurrences of sustained VT or VF in group 1 and group 2, respectively. Among patients with prior history of sustained VT/VF (n = 84; 48 in group 1 and 36 in group 2), 39.6% (19/48) and 13.9% (5/36) of the patients had recurrences of sustained VT or VF in group 1 and group 2, respectively. After univariate and multivariate Cox regression analysis in the subgroup with documented sustained VT (n = 84), the endocardial scar in the superior TV in the endocardium area was associated with VT/VF recurrence in the entire study population (HR: 3.596; 95% confidence interval [CI]: 1.412–9.160, *P* = .007; Supplemental Table 1) and in patients with endo-epicardial mapping (HR: 4.702, 95% CI: 1.676–13.193, *P* = .003, Supplemental Table 2).

## Discussion

### Main findings

The present study had several important findings. First, both endocardial and epicardial scars were more extensive in patients with ARVC and RV dysfunction. Second, the distribution of scars differs between ARVC patients with or without RV dysfunction. In the endocardium, there were more patients with scar involvement in the TV area and inferior wall in the RV dysfunction group than in the other group. In the epicardium, there were more patients with scar involvement in the inferior wall and fewer patients with scarring in the RVOT in the RV dysfunction group than in the other group. Third, the presence of endocardial superior TV scars was associated with long-term VT/VF recurrence.

### ARVC and the scar pattern

In patients with ARVC, the fibrofatty scar usually progresses from the epicardium toward the endocardium.^1^ In our study, the epicardial scar was more extensive than the endocardium in both groups, which is consistent with previous reports. The scar predominantly involves the RV free wall in patients with ARVC, which results in wall thinning and aneurysmal dilatation. The scar distribution is typically localized in the inflow tract (TV area), outflow tract, and apex.^3^^,^^18^ In the present study, no patient presented with scarring in the endocardial apex. In the epicardium, 4 patients had apical scar involvement with RV dysfunction. No scar involvement at the apex was observed in patients with preserved RV function.

**Figure 3:**
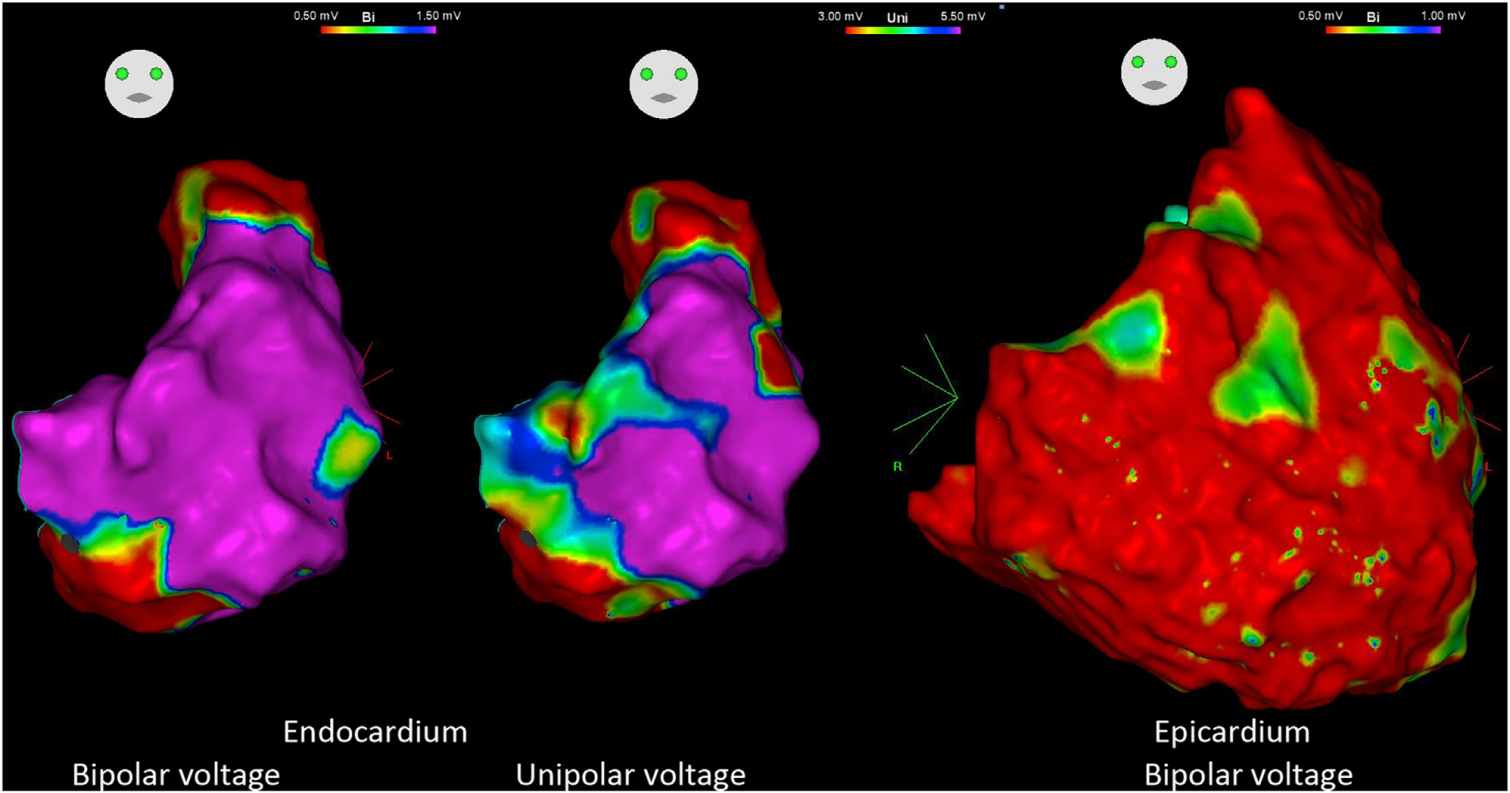
Example of group 1 patient with entire right ventricle (RV) epicardial scar. The endocardial bipolar voltage map (left image) shows a dense scar in the inferior tricuspid valve (TV) and inferior free wall. The endocardial unipolar voltage map (middle image) shows a comparable low-voltage zone in the same area and superior TV. The epicardial bipolar voltage map (right image) shows extensive scarring throughout the RV.

To the best of our knowledge, this is the first report describing a difference in scar distribution in ARVC patients with or without RV dysfunction. In patients with RV dysfunction, the scar was more dominant in the inferior portion and TV area. Conversely, the scar was more dominant in the superior portion of the patient without RV dysfunction. Our prior publication described the scar progression in patients with ARVC who underwent repeat procedures.^2^ In patients with recurrent VT, scar involvement tends to extend with the deterioration of RV systolic function. In our study cohort, 4 patients presented with homogeneous epicardial RV scarring and RV dysfunction (Figure 3). Patients with preserved RV systolic function may progress and present with more extensive scars and worsening RV dysfunction.

### Scar involvement and long-term recurrence

Considerable information has been published regarding risk stratification in patients with ARVC. The information was mostly the result of single-center reports and several small multicenter registries. In a previous study, the extent of electroanatomic scar on RV endocardial voltage mapping was associated with VT/VF recurrence.^19^^,^^20^ The RV dysfunction and LV dysfunction were associated with VT/VF events and adverse cardiovascular outcomes in previous studies.^21^, ^22^, ^23^ In our present study, LV dysfunction and extensive RV endocardial scarring were associated with the presence of RV dysfunction. Multivariate analysis showed that a scar involving the specific area (superior TV area) was independently associated with recurrence. Additionally, a longer activation time in the endocardium was also related to long-term recurrence (Supplemental Table 1). However, when we performed the subgroup analysis with the patient with endo-epicardial mapping, the statistical result became insignificant (Supplemental Table 2).

### Requirement of epicardial mapping/ablation

In our present study, more patients (35 [61.4%]) underwent epicardial mapping in group 1 in comparison to group 2 (14 [28.6%], *P* < .01). Additionally, the area with abnormal substrate was larger in group 1 patients in comparison to group 2 patients. Previous study suggested that patients with more advanced stage of ARVC tend to have more scar and less viable arrhythmogenic substrate in the epicardium owing to the progressive nature of ARVC.^10^ Therefore, the role of the epicardial approach might be less important in the advanced stage of ARVC. The finding was different from our results. Berruezo and colleagues^10^ defined that the advanced stage of ARVC was based on the substrate extension, which was different from our study. Further studies with more patients with ARVC are warranted to validate this result.

In our study, there was larger endocardial and epicardial scar area in the group 1 patients in comparison to group 2 patients. The extensive scar might indicate intramural wide-spreading fibrofatty infiltration and prohibit the energy penetration from the endocardial ablation.^24^ Therefore, the epicardial approach could be required to eliminate the intramural circuit in group 1 patients.

### Limitations

The present study had some limitations. First, some of the study population did not receive epicardial mapping. The results of the present study might be confounded by the retrospective nature of the study. In our study population, some patients were not indicated for an epicardial approach based on our methodology. Therefore, the information of epicardial substrate was not complete. Whether selective bias could confound the current results remains unknown, and further investigations are warranted to validate the generalizability of the present findings in a prospective cohort. Third, the presence of epicardial fat could interfere with the recognition scar within the epicardium. Fourth, we analyzed the scar distribution pattern, which might not indicate the area of slow conduction and the VT substrate for reentry arrhythmia.

## Conclusion

Patients with ARVC and RV dysfunction were associated with larger abnormal substrates in the endocardium and epicardium of the RV. The characteristics of scar distribution differed between ARVC patients with and without RV dysfunction. There were more scars involving the inferior portion and TV and fewer scars involving the RVOT in patients with RV dysfunction than in those without RV dysfunction. In the subgroup analysis of the patients with sustained VT, the presence of a scar in the superior TV of the endocardium could predict recurrence despite successful ablation.

## Perspectives

This study demonstrated the substrate characteristics in patients with arrhythmogenic right ventricular cardiomyopathy with or without right ventricular dysfunction. Diseased substrate involving the inflow tract or the tricuspid annulus and the inferior wall of the right ventricle was associated with the right ventricular dysfunction. In contrast, the diseased substrate involving the outflow tract was associated with the preserved right ventricular function. Catheter ablation was effective in eliminating the ventricular arrhythmia. However, the presence of a dense scar in the superior tricuspid valve was associated with recurrent ventricular tachycardia despite catheter ablation in the subgroup with sustained ventricular tachycardia before procedure.
