# Supplementary material for: Clinical significance of structural remodeling concerning substrate characteristics and outcomes in arrhythmogenic right ventricular cardiomyopathy
Source: Heart Rhythm O2. 2022 May 5;3(4):422–9. doi: 10.1016/j.hroo.2022.04.007 (PMC9463695; doi:10.1016/j.hroo.2022.04.007)
Supplement: Supplemental Tables 1 and 2 [file mmc1.docx]

**Clinical Significance of Structural Remodeling Concerning Substrate Characteristics and Outcomes in Arrhythmogenic Right Ventricular Cardiomyopathy**

Supplementary materials

Supplemental Table 1: Multivariate Analysis for Predictors of Recurrence with sustained VT/VF

Supplemental Table 2: Univariate Analysis for Predictors of Recurrence in patients with epicardial mapping

| **Supplemental Table 1. Multivariate Analysis for Predictors of Recurrence with sustained VT/VF (N=84)** | | | | | | |
| --- | --- | --- | --- | --- | --- | --- |
|  | Univariate analysis | | | Multivariate analysis | | |
|  | Crude HR | 95% CI | P value | Adjusted HR | 95% CI | P value |
| Age, yrs | 0.997 | 0.968-1.027 | 0.852 |  |  |  |
| Male | 2.426 | 0.966-6.094 | 0.059 | 0.890 | 0.251-3.162 | 0.857 |
| Hypertension | 1.910 | 0.862-4.231 | 0.111 | — | — | — |
| Diabetes | 0.884 | 0.119-6.572 | 0.904 | — | — | — |
| LVEF | 0.969 | 0.930-1.010 | 0.969 |  |  |  |
| RVEF | 0.972 | 0.941-1.003 | 0.072 | 1.003 | 0.960-1.047 | 0.899 |
| **Electrophysiological parameter** | | |  |  |  |  |
| Total activation time (ms) | 1.015 | 1.004-1.026 | 0.009 | 1.010 | 0.997-1.024 | 0.132 |
| Bipolar low voltage zone, % | 1.031 | 0.986-1.077 | 0.178 |  |  |  |
| Bipolar scar % | 1.045 | 0.972-1.123 | 0.231 |  |  |  |
| Unipolar LVZ % | 1.014 | 0.982-1.048 | 0.390 |  |  |  |
| **Endocardial scar segment** | | |  |  |  |  |
| RVOT | 0.889 | 0.410-1.929 | 0.766 | — | — | — |
| Superior free wall | 0.333 | 0.078-1.420 | 0.137 | — | — | — |
| Inferior free wall | 0.644 | 0.152-0.2734 | 0.551 | — | — | — |
| Superior TV | 4.787 | 2.137-10.723 | <0.001 | 3.596 | 1.412-9.160 | 0.007 |
| Inferior TV | 4.560 | 1.914-10.863 | 0.001 | 2.082 | 0.617-6.465 | 0.205 |
| *CI = confidence interval; LVEF = left ventricular ejection fraction; HR = hazard ratio; VT = ventricular tachycardia; TV = tricuspid valve; RVEF = right ventricular ejection fraction; RVOT = right ventricular outflow tract | | | | | | |

| **Supplemental Table 2. Univariate Analysis for Predictors of Recurrence in patients with epicardial mapping (N=49)** | | | | |
| --- | --- | --- | --- | --- |
|  | Univariate analysis | | |  |
|  | Crude HR | 95% CI | P value |  |
| Age, yrs | 0.985 | 0.943-1.029 | 0.507 |  |
| Male | 2.896 | 0.655-12.798 | 0.161 |  |
| Hypertension | 2.019 | 0.746-5.462 | 0.166 |  |
| Diabetes | 0.839 | 0.110-6.375 | 0.865 |  |
| LVEF | 0.968 | 0.917-1.022 | 0.239 |  |
| RVEF | 0.986 | 0.945-1.029 | 0.512 |  |
| **Electrophysiological parameter** |  |  |  |  |
| Total activation time (ms) | 1.011 | 0.993 -1.030 | 0.226 |  |
| Bipolar low voltage zone, % | 1.026 | 0.967-1.089 | 0.395 |  |
| Bipolar scar % | 1.067 | 0.967-1.167 | 0.152 |  |
| Unipolar LVZ % | 0.980 | 0.932-1.032 | 0.448 |  |
| **Endocardial scar segment** | — | — | — |  |
| RVOT | 0.829 | 0.311-2.211 | 0.707 |  |
| Superior free wall | 0.470 | 0.101-21.205 | 0.696 |  |
| Inferior free wall | 0.220 | 0.029-1.671 | 0.143 |  |
| Superior TV | 4.702 | 1.676-13.193 | 0.003 |  |
| Inferior TV | 1.885 | 0.652-5.448 | 0.242 |  |
| Epicardial scar segment | — | — | — |  |
| RVOT | 0.759 | 0.282-2.043 | 0.586 |  |
| Superior free wall | 0.420 | 0.001-65.655 | 0.397 |  |
| Inferior free wall | 1.119 | 0.419-2.989 | 0.822 |  |
| Superior TV | 1.213 | 0.449-3.277 | 0.703 |  |
| Inferior TV | 1.331 | 0.428-4.138 | 0.622 |  |
| *VT = ventricular tachycardia; TV = tricuspid valve; LVEF = left ventricular ejection fraction; RVEF = right ventricular ejection fraction; RVOT = right ventricular outflow tract | | | | |
